# Clinical outcomes and Oncotype DX Breast Recurrence Score® in early‐stage BRCA‐associated hormone receptor‐positive breast cancer

**DOI:** 10.1002/cam4.4566

**Published:** 2022-02-06

**Authors:** Rachel M. Layman, Heather Lin, Angelica M. Gutierrez Barrera, Meghan S. Karuturi, Clinton Yam, Banu K. Arun

**Affiliations:** ^1^ Department of Breast Medical Oncology University of Texas MD Anderson Cancer Center Houston Texas USA; ^2^ Department of Biostatistics University of Texas MD Anderson Cancer Center Houston Texas USA

**Keywords:** *BRCA1*, *BRCA2*, breast cancer, estrogen receptor, gene expression profiling, survival analysis

## Abstract

**Background:**

BRCA‐associated breast cancers tend to have distinctive features compared to sporadic breast cancers; further characterization can aid in optimizing treatment.

**Methods:**

The study evaluated a patient cohort with early‐stage estrogen receptor positive, HER2 negative invasive breast cancer who had Oncotype DX Breast Recurrence Score**®** analysis and genetic testing for hereditary breast and ovarian cancer syndrome. Data on patients and their breast cancers with outcomes were collected and analyzed.

**Results:**

745 patients were included, of whom 33 had pathogenic BRCA mutations (8 *BRCA1*, 25 *BRCA2*). Patients with BRCA mutations were younger and received more adjuvant chemotherapy, but less endocrine therapy and radiation therapy. BRCA‐associated breast cancers had less progesterone receptor expression, higher nuclear grade, and higher Oncotype DX Breast Recurrence Scores**®** with median Recurrence Score**®** 29, compared to 16 in cancers without mutations (*p* < 0.0001). Breast cancer recurrence developed in 18% of patients with BRCA mutations and 9% of patient without mutations, although multivariate analysis of relapse‐free survival was not significant, HR 1.519 (95% confidence interval [CI] 0.64–3.58; *p* = 0.3401). After adjusting for Recurrence Score**®**, overall survival by BRCA status was improved HR 0.448 (95% CI 0.06–3.34; *p* = 0.4333).

**Conclusions:**

BRCA‐associated early‐stage hormone receptor‐positive breast cancers have higher Oncotype DX Breast Recurrence Score**®** compared to those without mutations. BRCA status did not significantly impact relapse‐free survival and overall survival. Larger clinical trials are needed to further assess the findings, and if confirmed, could impact clinical management of BRCA‐associated breast cancers.

## Graphical abstract

The study evaluated a patient cohort with early‐stage estrogen receptor positive, HER2‐negative invasive breast cancer who had Oncotype DX Breast Recurrence Score® analysis and genetic testing for hereditary breast and ovarian cancer syndrome. BRCA‐associated breast cancers had less progesterone receptor expression, higher nuclear grade, and higher Oncotype DX Breast Recurrence Scores® with median Recurrence Score® 29, compared to 16 in cancers without mutations (*p* < 0.0001). After adjusting for Recurrence Score®, overall survival by BRCA status was improved HR 0.448 (95% CI 0.06–3.34; *p* = 0.4333).

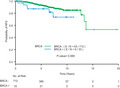

## 1 INTRODUCTION

While most breast cancers are sporadic, approximately 20% are considered to be familial and about 5%–10% develop due to a single mutation in a breast cancer susceptibility gene.^1^ Mutations in *BRCA1* and *BRCA2*, which cause Hereditary Breast and Ovarian Cancer Syndrome (HBOC), account for around 80% of the 5%–10% of breast cancer cases related to rare mutations.^1^, ^2^ Individuals with *BRCA1*/*BRCA2* mutations have up to a 70% life‐time risk of developing breast cancer and up to 45% risk of developing ovarian cancer.^1^, ^2^, ^3^

Several small studies have reported differences in pathologic and clinical characteristics of both HBOC and non‐*BRCA* familial breast cancer. Breast carcinomas arising from a *BRCA1* mutation have been shown to be somewhat distinctive, more often high grade and negative for estrogen receptor (ER), progesterone receptor (PR), and human epidermal growth factor receptor 2 (Her2).^4^, ^5^ *BRCA1‐*associated breast cancers are more likely to be invasive ductal type versus invasive lobular, tend to have a high mitotic rate and more frequently manifest a pattern of lymphocytic infiltration.^4^, ^6^ In contrast, *BRCA2*‐associated breast cancers are not well‐defined overall.^7^ *BRCA2*‐associated breast cancers are more likely to be hormone receptor positive (HR+) and have lower tumor grade and mitotic counts compared to *BRCA1*‐associated breast cancers.^4^, ^6^, ^8^ Women with *BRCA2* mutations have an increased risk of invasive lobular breast cancer, as opposed to *BRCA1* mutation carriers.^9^ While the observations would be expected to have strong implications on prognosis, data on BRCA‐associated (*BRCAm*) breast cancers are inconsistent.^10^ Most studies describing characteristics of *BRCAm* cancers are small and descriptive involving data from multiple institutions.

The Oncotype DX Breast Recurrence Score® test (Exact Sciences Corporation) is a 21 gene expression assay that provides prognostic and predictive information for patients with early‐stage HR + breast cancer. Higher Recurrence Score**®** results (RS) are associated with worse prognosis and greater benefit from chemotherapy.^11^, ^12^. As *BRCA*m breast cancers appear to have inherent differences from those without *BRCA* mutations (*BRCA*wt), it is important to understand the interaction between *BRCA*m breast cancers and RS, as this can impact systemic therapy treatment decision making.

Herein, we report the outcomes and characteristics of patients and breast cancers, including RS, in a large cohort of patients with *BRCA*m and *BRCA*wt early‐stage HR+, HER2‐negative breast cancer.

## 2 MATERIALS AND METHODS

### 2.1 Patient population

Patients with invasive HR+, HER2‐negative breast cancers who have had Oncotype DX Breast Recurrence Score**®** testing and who underwent genetic testing in the Breast Medical Oncology and the Breast Clinical Cancer Genetics Clinic at the University of Texas MD Anderson Cancer Center (MDACC) were identified from the prospectively maintained Breast Cancer and the Genetics research registries.

For patients diagnosed with more than one invasive breast cancer, only the first cancer was included in the data and analysis. Patients with ER‐negative or HER2‐positive breast cancers were excluded.

### 2.2 Assessment

The following data were collected and analyzed as part of this study: demographic information, reproductive history (age at menarche, pregnancy history, and oral contraceptive and hormonal therapy use), genetic testing results, tumor characteristics (including gene profiling, Oncotype DX Breast Recurrence Score**®**), family history, cancer treatment, chemotherapy treatments, and response to chemotherapy, recurrence/metastasis information and survival data. Data collection was performed through August 28, 2020.

### 2.3 Informed consent and data confidentiality procedures

Patients at MDACC had previously provided consent for their data to be used for future research on protocols LAB03‐0479, LAB99‐402, and 2003–321. In addition, waiver of consent for the study was provided by the MDACC Institutional Review Board since this retrospective study involves no more than minimal risk, does not adversely affect the rights or welfare of the patients, and otherwise could not reasonably be carried out.

The information was kept in a database on a password‐protected computer in a secure office and the data was only accessible to the study investigators. After the clinical information was obtained, all patient identifying information were deleted and replaced by unique study numbers in the analytic file. No patient identifiers were used when analyzing or reporting the data and were deleted after data analysis.

## 3 STATISTICAL ANALYSIS

The study objectives were to assess the associations between BRCA status and recurrence‐free survival (RFS) and overall survival (OS) among all patients, and to assess the association between BRCA status and the RS among the patients who underwent Oncotype DX Breast Recurrence Score**®** testing.

Univariate analyses were performed to evaluate the associations of patient's demographic and clinical characteristics, including the RS, with BRCA mutation status, using chi‐square tests for categorical variables and t tests/ANOVA or the counterparts of the non‐parametric approaches (Wilcoxon rank‐sum or Kruskal–Wallis for continuous variables).^13^ The distributions of RFS and OS were estimated by the Kaplan–Meier method.^14^ Log‐rank test^15^ was performed to test the differences in survival between groups. Regression analyses of survival data based on the Cox proportional hazards model^16^ were conducted on RFS defined as from the time of diagnosis to the time of local recurrence, distant metastasis, or death, whichever occurred first, and OS defined as from the time of diagnosis to the time of death. Time was censored at the last contact at which the patient was known to be recurrence‐free for RFS and the last time the patient was known to be alive for OS. SAS version 9.4 and S‐Plus version 8.2 were used to carry out the computations for all analyses.

## 4 RESULTS

### 4.1 Patients

A total of 745 patients met the eligibility criteria and were included in the analysis. Patient characteristics are shown in Table 1. Thirty‐three (4.4%) patients had identified pathogenic *BRCA1* (8) or *BRCA2* (25) germline mutations. The majority of patients (57%) were under the age of 50 at breast cancer diagnosis. A family history of breast cancer was reported in 67% of patients and 16% had a family history of ovarian cancer.

**TABLE 1:** Patient characteristics

Patient characteristics (*n* = 745)		
		*n* (%)
BRCA status	*BRCA1*	8 (1.1)
	*BRCA2*	25 (3.3)
	Negative	712 (95.6)
Age at diagnosis	≤50	423 (56.8)
	>50	322 (43.2)
Race	White	519 (69.7)
	Black	36 (4.8)
	Hispanic	126 (16.9)
	Asian	52 (7.0)
	Other	12 (1.6)
Breast cancer, 1st degree relative	0	486 (65.2)
	≥1	199 (26.7)
	Unknown	60 (8.1)
Breast Cancer Family Hx	0	182 (24.4)
	≥1	501 (67.3)
	Unknown	62 (8.3)
Ovarian cancer, 1st degree relative	0	649 (87.1)
	1	36 (4.8)
	Unknown	60 (8.1)
Ovarian Cancer Family Hx	0	564 (75.7)
	≥1	119 (16.0)
	Unknown	62 (8.3)

Abbreviation: Hx, history.

All patients had ER+ breast cancer, and most were also PR positive. Most cancers were Stage I (66%) with only 15% having documented positive lymph nodes. Over 90% of the patients received endocrine therapy, while approximately 1/3 received chemotherapy. Cancer characteristics and treatment are summarized in Table 2.

**TABLE 2:** Cancer characteristics and treatment

(*n* = 745)		*n* (%)
Estrogen receptor	POS	745 (100)
	NEG	0 (0)
Progesterone receptor	POS	668 (89.7)
	NEG	76 (10.2)
	Unknown	1 (0.1)
HER2 status	POS	0 (0.0)
	NEG	312 (41.9)
	Unknown	433 (58.1)
Stage	I	492 (66.04)
	II	234 (31.41)
	III	13 (1.74)
	Unknown	6 (0.81)
T stage	T0	1 (0.1)
	T1	542 (72.8)
	T2	176 (23.6)
	T3	19 (2.6)
	T4b	1 (0.1)
	Unknown	6 (0.8)
N stage	N0	624 (83.7)
	N1	107 (14.4)
	N2	6 (0.8)
	NX	2 (0.3)
	Unknown	6 (0.8)
Nuclear grade	I	90 (12.1)
	II	463 (62.1)
	III	164 (22.0)
	Unknown	28 (3.8)
Lymphatic invasion	POS	113 (15.2)
	NEG	599 (80.4)
	Unknown	33 (4.4)
Vascular invasion	POS	112 (15.0)
	NEG	600 (80.6)
	Unknown	33 (4.4)
Surgical margins	Negative	683 (91.7)
	Close (<2 mm)	15 (2.0)
	Positive	5 (0.7)
	Unknown	42 (5.6)
Neoadjuvant chemo	Yes	17 (2.3)
	No	728 (97.7)
Adjuvant chemo	Yes	250 (33.6)
	No	495 (66.4)
Neoadjuvant Endocrine Tx	Yes	10 (1.3)
	No	735 (98.7)
Adjuvant Endocrine Tx	Yes	680 (91.3)
	No	65 (8.7)
Radiation Tx	Yes	353 (47.4)
	No	392 (52.6)

Abbreviations: Chemo, chemotherapy; NEG, negative; POS, positive; Tx, treatment.

### 4.2 Association of BRCA status on patient and cancer characteristics

No statistically significant difference in baseline patient characteristics was observed between *BRCAm* or *BRCAwt* patients (*p* ≥ 0.21), however, there were non‐significant numerical differences. *BRCAm* patients were younger with a median age at diagnosis 45 compared to 48 years, had more diverse race, and more family members with ovarian cancer. Similarly, tumor characteristics were not significantly different (*p* ≥ 0.066). More patients with BRCA mutations had PR‐negative cancers, 18% versus 10%, and higher nuclear grade. Only 1 *BRCAm* cancer was nuclear grade 1. Conversely, treatment differed significantly among the two groups. Over half of the *BRCAm* patients received chemotherapy, compared to approximately 1/3 of *BRCAwt* patients (*p* = 0.009). *BRCAm* patients were less likely to receive adjuvant endocrine therapy (82% vs. 92%, *p* = 0.0049) or radiation therapy (24% vs. 49%, *p* = 0.0065). See Table 3.

**TABLE 3:** Association between BRCA status and patient characteristics, cancer characteristics, and treatment^a^

		BRCA		*p*‐value
		Negative	Positive	
Age at diagnosis	≤50	401 (56.3%)	22 (66.7%)	0.24
	>50	311 (43.7%)	11 (33.3%)	
Race	White	500 (70.2%)	19 (57.6%)	0.22
	Hispanic	120 (16.9%)	6 (18.2%)	
	Black	33 (4.6%)	3 (9.1%)	
	Asian	48 (6.7%)	4 (12.1%)	
	Other	11 (1.5%)	1 (3%)	
Breast cancer, 1st degree relative	0	471 (71.1%)	15 (65.2%)	0.54
	≥1	191 (28.9%)	8 (34.8%)	
Breast Cancer Family Hx	0	174 (26.4%)	8 (34.8%)	0.34
	≥1	486 (73.6%)	15 (65.2%)	
Ovarian cancer, 1st degree relative	0	628 (94.9%)	21 (91.3%)	0.27
	≥1	34 (5.1%)	2 (8.7%)	
Ovarian Cancer Family Hx	0	547 (82.9%)	17 (73.9%)	0.2651
	≥1	113 (17.1%)	6 (26.1%)	
Progesterone receptor	POS	641 (90.2%)	27 (81.8%)	0.12
	NEG	70 (9.8%)	6 (18.2%)	
Stage	I	468 (66.3%)	24 (72.7%)	0.40
	II	226 (32.0%)	8 (24.2%)	
	III	12 (1.7%)	1 (3%)	
T stage	T0	0 (0%)	1 (3%)	0.24^b^
	T1	515 (72.9%)	27 (81.8%)	
	T2	172 (24.4%)	4 (12.1%)	
	T3	18 (2.5%)	1 (3%)	
	T4b	1 (0.1%)	0 (0%)	
N stage	N0	597 (84.6%)	27 (81.8%)	0.33^c^
	N1	103 (14.6%)	4 (12.1%)	
	N2	5 (0.7%)	1 (3%)	
	NX	1 (0.1%)	1 (3%)	
Nuclear grade	I	89 (13.0%)	1 (3.1%)	0.066
	II	444 (64.8%)	19 (59.4%)	
	III	152 (22.2%)	12 (37.5%)	
Lymphatic invasion	NEG	573 (84.1%)	26 (83.9%)	1.00
	POS	108 (15.9%)	5 (16.1%)	
Vascular invasion	NEG	574 (84.3%)	26 (83.9%)	1.00
	POS	107 (15.7%)	5 (16.1%)	
Adjuvant Chemo	Y	232 (32.6%)	18 (54.5%)	**0.0090**
	N	480 (67.4%)	15 (45.5%)	
Adjuvant Endocrine Tx	Y	653 (91.7%)	27 (81.8%)	**0.0489**
	N	59 (8.3%)	6 (18.2%)	
Radiation Tx	Y	345 (48.5%)	8 (24.2%)	**0.0065**
	N	367 (51.5%)	25 (75.8%)	

The bold values indicate statistically significant.

Abbreviations: Chemo, chemotherapy; Hx, history; NEG, negative; POS: positive; Tx, treatment.

^a^ Missing or unknown data was excluded from this analysis.

^b^ The patients with T0 or T4b tumor were not included in the comparison between the BRCA positive and BRCA negative patients.

^c^ The two patients with NX tumor were not included in the comparison between the BRCA positive and BRCA negative patients.

### 4.3 Recurrence Score® results

The impact of patient and tumor characteristics on the RS was evaluated (Table 4). *BRCAm* patients had significantly higher RS than those without mutation. The median RS was 29 with *BRCAm* and 16 with *BRCAwt*, *p* < 0.0001. Cancers that were PR negative or with higher nuclear grade also had higher RS (*p* < 0.0001).

**TABLE 4:** Association between patient characteristics and Oncotype DX Breast Recurrence Score®^a^

		*n*	Oncotype DX recurrence score			*p*‐value
			Range	Mean (SD)	Median	
BRCA	NEG	712	0–71	18.2 (10.6)	16.0	**<0.0001**
	POS	33	10–60	28.0 (12.0)	29.0	
Lymphatic invasion	NEG	599	0–71	18.8 (11.0)	16.0	0.46
	POS	113	0–47	17.2 (8.4)	16.0	
Vascular invasion	NEG	600	0–71	18.8 (11.0)	16.0	0.53
	POS	112	0–47	17.3 (8.4)	16.5	
Nuclear grade	I	90	0–35	14.7(6.5)	13.5	**<0.0001**
	II	463	0–52	16.0 (7.7)	16.0	
	III	164	5–71	28.0 (14.0)	26.0	
Pathologic stage	I	492	0–56	18.5 (10.1)	16.0	0.54
	II	234	0–71	18.8 (11.9)	16.0	
	III	13	13–44	20.7 (9.0)	18.0	
T stage	T0^b^	1	26–26	26.0 (NA)	26.0	0.8^a^
	T1	542	0–56	18.4 (9.9)	16.0	
	T2	176	0–71	19.6 (13.0)	16.0	
	T3	19	0–32	15.9 (8.4)	17.0	
	T4b^b^	1	16–16	16.0 (NA)	16.0	
N stage	N0	624	0–71	18.8 (11.0)	16.0	0.35^b^
	N1	107	0–38	17.0 (8.4)	17.0	
	N2	6	13–44	23.5 (11.3)	22.0	
	NX^c^	2	17–20	18.5 (2.1)	18.5	
Progesterone receptor	NEG	76	11–67	33.3 (13.9)	31.0	**<0.0001**
	POS	668	0–71	17.0 (9.0)	16.0	
Age at diagnosis	≤50	423	0–67	18.2 (10.2)	16.0	0.28
	>50	322	0–71	19.2 (11.5)	17.0	
Race	White	519	0–67	18.3 (10.5)	16.0	0.097
	Hispanic	126	0–71	19.1 (12.0)	17.0	
	Black	36	1–56	22.9 (12.2)	22.0	
	Asian	52	0–40	18.4 (9.0)	16.5	
	Other	12	1–42	18.2 (12.1)	16.5	
Breast cancer, 1st degree relative	0	486	0–67	18.8 (10.6)	17.0	0.37
	≥1	199	0–71	18.5 (11.8)	16.0	
Breast cancer Family Hx	0	182	0–67	18.3 (10.3)	16.5	0.76
	≥1	501	0–71	18.9 (11.2)	16.0	
Ovarian cancer, 1st degree relative	0	649	0–71	18.7 (10.9)	16.0	0.78
	≥1	36	1–60	18.9 (11.1)	17.0	
Ovarian cancer Family Hx	0	564	0–71	18.7 (10.9)	16.0	0.41
	≥1	119	0–60	19.1 (11.1)	17.0	

The bold values indicate statistically significant.

Abbreviations: Hx, history; NEG, negative; POS, positive; SD, standard deviation.

^a^ The patients with T0 or T4b tumor were not included in the comparison between the BRCA positive and BRCA negative patients.

^b^ The two patients with NX tumor were not included in the comparison between the BRCA positive and BRCA negative patients.

^c^ Missing or unknown data was excluded from this analysis.

### 4.4 Patient outcomes

**FIGURE 1:**
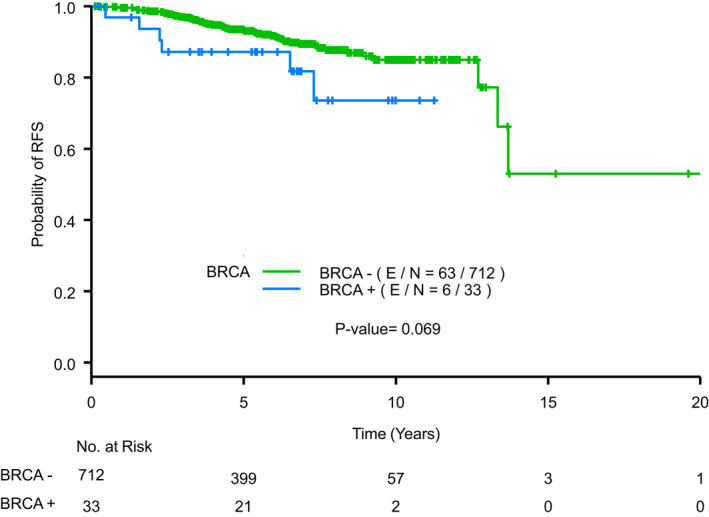
Recurrence‐free survival by BRCA mutation status legend: Kaplan–Meier curve displaying recurrence‐free survival (RFS) by the presence or absence of a germline *BRCA1* or *BRCA2* mutation. E/N, number of events/total number of patients

**FIGURE 2:**
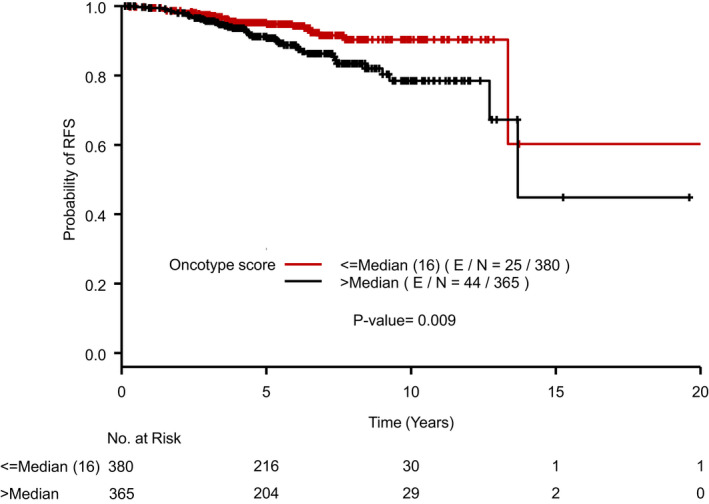
Recurrence‐free survival by oncotype DX recurrence score. Kaplan–Meier curve displaying recurrence‐free survival (RFS) by high or low Oncotype DX Breast Recurrence Score® with median of 16 used as the cutoff. E/N, number of events/total number of patients

Patients in the study were followed for median of 5.9 years. RFS was inferior in *BRCAm* patients with RFS events in 6/33 (18.18%) compared to *BRCAwt* patients who had 63/712 (8.85%) events (Figure 1). However, in multivariate analysis, adjusting for RS and receipt of endocrine therapy, RFS by BRCA status was not statistically significant with HR 1.52 (95% confidence interval [CI] 0.64–3.58; *p* = 0.3401). Lower RS was associated with improved RFS (Figure 2).

**FIGURE 3:**
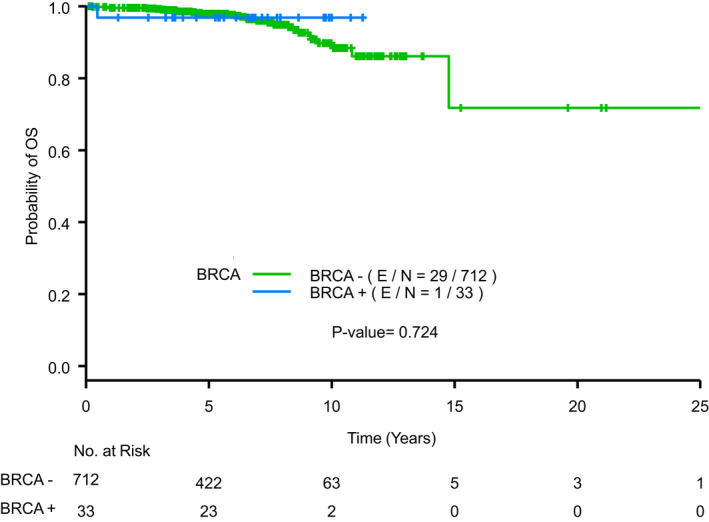
Overall survival by BRCA mutation status. Kaplan–Meier curve displaying overall survival (OS) by the presence or absence of a germline *BRCA1* or *BRCA2* mutation. E/N, number of events/total number of patients

**FIGURE 4:**
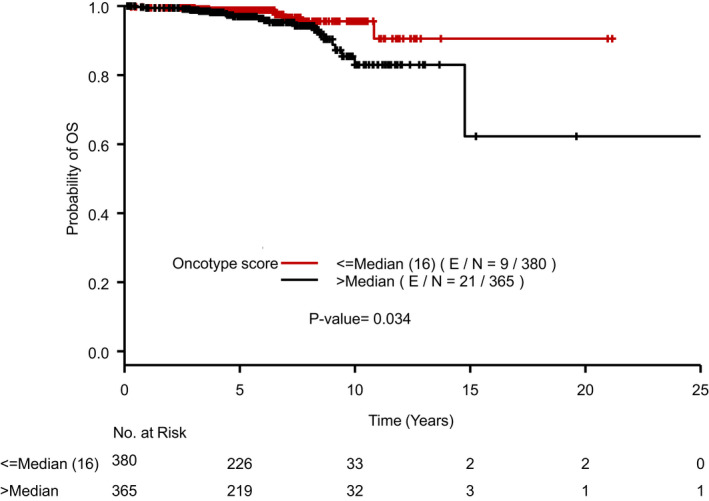
Overall survival (OS) by Oncotype DX Recurrence Score. Legend: Kaplan–Meier curve displaying overall survival (OS) by high or low Oncotype DX Breast Recurrence Score® with median of 16 used as the cutoff. E/N, number of events/total number of patients

*BRCAm* patients had a small improvement in OS with 1/33 (3.03%) deaths compared to *BRCAwt* patients who had 29/712 (4.07%) deaths (Figure 3). On univariate analysis, only RS had a statistically significant impact on OS. After adjusting for RS, the difference in OS by BRCA status was not statistically significant with HR 0.448 (95% CI 0.06–3.34, *p* = 0.4333). However, lower RS was again associated with improved OS (Figure 4).

## 5 DISCUSSION

This study evaluated differences between early‐stage HR+, HER2‐negative breast cancers among patients with and without germline BRCA mutations. We demonstrated that *BRCAm* breast cancers are associated with higher Oncotype DX Breast Recurrence Scores**®**, which is consistent with other reports.^17^, ^18^, ^19^ To our knowledge, this is the first report that also includes survival data. In the overall population, lower RS is associated with improved OS. Of interest, *BRCAm* patients had a small numerical improvement in OS compared to *BRCAwt* patients. However, the improved OS was not statistically significant after adjusting for RS, demonstrating an HR of 0.448 but a wide confidence interval. The small number of *BRCAm* patients may have affected the results.

Use of RS to aid in clinical decision making is part of routine clinical care and has been validated as both a prognostic and predictive tool for early‐stage HR+ breast cancer.^11^, ^12^ The positive association of higher RS in *BRCAm* cancers provides further evidence that *BRCAm* breast cancers have distinct characteristics compared to *BRCAwt*. Cancers with *BRCA1* and *BRCA2* mutations have homologous recombination deficiency resulting in error‐prone repair of double‐strand DNA breaks and overall genetic instability.^20^ Accordingly, BRCA‐associated breast cancers are more sensitive to DNA‐damaging agents^21^ and may be biologically primed to receive greater benefit from adjuvant chemotherapy. Also, genetic instability may render the cancers more likely to develop resistance mechanisms to endocrine therapy. Better understanding of the biology of *BRCAm* cancers is crucial to optimizing management and thereby improving outcomes.

The data suggest that HR+ *BRCAm* cancers are more likely to be PR negative and have a higher nuclear grade, properties that may suggest more aggressive cancers that are less responsive to endocrine therapy. Similarly, in the entire study population, PR negative and higher nuclear grade cancers had higher RS. Our study demonstrated that lower RS is associated with significantly better outcomes, including RFS and OS. Since patients with high RS breast cancers benefit from chemotherapy, it is possible that *BRCAm* cancers may be more likely to benefit from chemotherapy. If this finding is confirmed, it would have important implications for adjuvant treatment decision making for *BRCAm* patients. Treating physicians may be more inclined to order Oncotype DX Breast Recurrence Score**®** testing before making the decision to omit chemotherapy for *BRCAm* breast cancers. To our knowledge, analysis of the utilization of RS in BRCAm patients is not available, however, such data would be of interest.

The study included patients with early‐stage HR+ breast cancer who had genetic testing. Of the 745 patients included in the patient cohort, only 33 had a *BRCA1* or *BRCA2* mutation. Given the propensity for triple‐negative breast cancer with *BRCA1* mutations, as anticipated, most of the identified mutations were in *BRCA2*; therefore, the findings may be less applicable to *BRCA1*‐associated breast cancers. The treatments administered differed significantly in patients with BRCA mutations compared to those without. Chemotherapy was administered more frequently in *BRCAm* patients, which is consistent with higher observed RS. However, endocrine therapy was given less often. It is possible that *BRCAm* breast cancers, especially those that are *BRCA1*, had lower levels of ER expression impacting the decision to proceed with endocrine therapy, however, this data is not available. *BRCAm* patients also received less radiation. While the surgical procedure performed for each patient is not known, it is likely that *BRCAm* patients were more inclined to choose mastectomy over breast conservation given the higher risk for second primary breast cancer. Most of the breast cancers were lymph node‐negative stage I or stage II, which would not require radiation if mastectomy was performed.

Despite the large study cohort, the study results are limited by the small number of patients with BRCA mutations. The study population is composed of patients who underwent a genetics evaluation, and as such, the patients are young with more than half under the age of 50, and most patients had a family history of breast cancer. While the results may not be applicable to the average woman with HR+ breast cancer, the *BRCAwt* population in our study is likely to be better matched to the *BRCAm* patients than the general early‐stage HR+ population. Additionally, intensified screening, such as breast MRI, is recommended for BRCAm patients per NCCN guidelines,^22^ and it is possible that this additional screening could have confounded the results. The small number of *BRCAm* patients may have limited the ability to detect differences in some of the variables evaluated as many were numerically different but not statistically significant.

Even with the limitations of a small *BRCAm* cohort, a statistically significant and clinically relevant increase in RS was detected in patients with *BRCA1*/*BRCA2* mutations compared to those without and is substantiated by other reports.^17^, ^18^, ^19^ This consequential finding should be further evaluated in larger studies. Given the biological differences between *BRCAm* and *BRCAwt* breast cancers, there is also concern that *BRCAm* patients may have a worse prognosis without chemotherapy even with a low RS, but currently, available data do not clearly validate this theory.^17^, ^23^ Larger studies to evaluate if RS in *BRCAm* patients provides prognostic and predictive value similar to that observed in the general population are needed to further optimize therapy.

## CONFLICT OF INTEREST

Rachel M. Layman: Research support paid to the institution from Eli Lilly, Novartis, Pfizer, GlaxoSmithKline, Puma, Zentalis, and Celcuity. Consulting fees from Eli Lilly, Novartis, Pfizer and Celcuity, Heather Lin: nothing to disclose, Angelica M. Gutierrez Barrera: nothing to disclose, Meghan Sri Karuturi: Consulting fees from Eli Lilly and Pfizer, Clinton Yam: Research support paid to the institution from Amgen, Merck, Genentech, and GlaxoSmithKline, Banu K. Arun: Research paid to the institution from Astra Zeneca, AbbVie, Invitae. Nonpaid steering committee member for AbbVie.

## AUTHOR CONTRIBUTIONS

Rachel M. Layman: Data analysis and interpretation, writing – original draft, and writing—review and editing. Heather Lin: Data acquisition, statistical analysis, data interpretation, writing—original draft, and writing—review and editing. Angelica M. Gutierrez Barrera: Design, data acquisition, data interpretation, writing—review and editing. Meghan Sri Karuturi: Data interpretation, writing—review and editing. Clinton Yam: Data interpretation, writing—review and editing. Banu K. Arun: Conception and design, data acquisition, data analysis and interpretation, writing—review and editing.

## PRECIS FOR USE IN THE TABLE OF CONTENTS

This study demonstrates that early‐stage BRCA‐associated hormone receptor‐positive breast cancers have higher Oncotype DX Breast Recurrence Score® results, less progesterone receptor expression and higher nuclear grade compared to those without BRCA mutations, however, relapse‐free survival and overall survival were not significantly different when adjusting for Recurrence Score**®**. Further studies are warranted to further understand the impact of germline BRCA mutations on tumor characteristics and outcomes to further guide optimal selection of adjuvant systemic therapy in this patient population.

## Data Availability

Data sharing is not applicable to this article as no new data were created or analyzed in this study.

## References

[1] Arun BK , Strong LC . Breast cancer genetic syndromes. In: Singletary SER GL , Hortobagyi GN , eds. Advanced Therapy of Breast Disease. BC Decker, Inc; 2004:75‐83.

[2] Stoppa‐Lyonnet D , Ansquer Y , Dreyfus H , et al. Familial invasive breast cancers: worse outcome related to BRCA1 mutations. J Clin Oncol. 2000;18:4053‐4059. 11118466 10.1200/JCO.2000.18.24.4053

[3] Kuchenbaecker KB , Hopper JL , Barnes DR , et al. Risks of breast, ovarian, and contralateral breast cancer for BRCA1 and BRCA2 mutation carriers. JAMA. 2017;317:2402‐2416. 28632866 10.1001/jama.2017.7112

[4] Chappuis PO , Nethercot V , Foulkes WD . Clinico‐pathological characteristics of BRCA1‐ and BRCA2‐related breast cancer. Semin Surg Oncol. 2000;18:287‐295. 10805950 10.1002/(sici)1098-2388(200006)18:4<287::aid-ssu3>3.0.co;2-5

[5] Atchley DP , Albarracin CT , Lopez A , et al. Clinical and pathologic characteristics of patients with BRCA‐positive and BRCA‐negative breast cancer. J Clin Oncol. 2008;26:4282‐4288. 18779615 10.1200/JCO.2008.16.6231 PMC6366335

[6] Lakhani SR , Jacquemier J , Sloane JP , et al. Multifactorial analysis of differences between sporadic breast cancers and cancers involving BRCA1 and BRCA2 mutations. J Natl Cancer Inst. 1998;90:1138‐1145. 9701363 10.1093/jnci/90.15.1138

[7] Da Silva L , Lakhani SR . Pathology of hereditary breast cancer. Mod Pathol. 2010;23(Suppl 2):S46‐S51. 20436502 10.1038/modpathol.2010.37

[8] Armes JE , Trute L , White D , et al. Distinct molecular pathogeneses of early‐onset breast cancers in BRCA1 and BRCA2 mutation carriers: a population‐based study. Cancer Res. 1999;59:2011‐2017. 10213514

[9] Yadav S , Hu C , Nathanson KL , et al. Germline pathogenic variants in cancer predisposition genes among women with invasive lobular carcinoma of the breast. J Clin Oncol. 2021;39:3918‐3926. 34672684 10.1200/JCO.21.00640 PMC8660003

[10] Bordeleau L , Panchal S , Goodwin P . Prognosis of BRCA‐associated breast cancer: a summary of evidence. Breast Cancer Res Treat. 2010;119:13‐24. 19789974 10.1007/s10549-009-0566-z

[11] Paik S , Tang G , Shak S , et al. Gene expression and benefit of chemotherapy in women with node‐negative, estrogen receptor‐positive breast cancer. J Clin Oncol. 2006;24:3726‐3734. 16720680 10.1200/JCO.2005.04.7985

[12] Sparano JA , Gray RJ , Makower DF , et al. Adjuvant chemotherapy guided by a 21‐gene expression assay in breast cancer. N Engl J Med. 2018;379:111‐121. 29860917 10.1056/NEJMoa1804710 PMC6172658

[13] Woolson RF , Clarke WR . Statistical Methods for the Analysis of Biomedical Data. 2nd ed. John Wiley & Sons; 2002.

[14] Kaplan EL , Meier P . Nonparametric estimator from incomplete observations. J Am Stat Assoc. 1958;53:457‐481.

[15] Mantel N . Evaluation of survival data and two new rank order statistics arising in its consideration. Cancer Chemother Rep. 1966;50:163‐170. 5910392

[16] Cox DR . Regression models and life tables (with discussion). J R Statist Soc B. 1972;34:187‐220.

[17] Blanter J , Zimmerman B , Tharakan S , Ru M , Cascetta K , Tiersten A . BRCA mutation association with recurrence score and discordance in a large oncotype database. Oncology. 2020;98:248‐251. 31962330 10.1159/000504965

[18] Halpern N , Sonnenblick A , Uziely B , et al. Oncotype DX recurrence score among BRCA1/2 germline mutation carriers with hormone receptors positive breast cancer. Int J Cancer. 2017;140:2145‐2149. 28120435 10.1002/ijc.30616

[19] Shah PD , Patil S , Dickler MN , Offit K , Hudis CA , Robson ME . Twenty‐one‐gene recurrence score assay in BRCA‐associated versus sporadic breast cancers: differences based on germline mutation status. Cancer. 2016;122:1178‐1184. 26859126 10.1002/cncr.29903

[20] Turner N , Tutt A , Ashworth A . Hallmarks of 'BRCAness' in sporadic cancers. Nat Rev Cancer. 2004;4:814‐819. 15510162 10.1038/nrc1457

[21] Kennedy RD , Quinn JE , Mullan PB , Johnston PG , Harkin DP . The role of BRCA1 in the cellular response to chemotherapy. J Natl Cancer Inst. 2004;96:1659‐1668. 15547178 10.1093/jnci/djh312

[22] Daly MB , Pal T , Berry MP , et al. Genetic/familial high‐risk assessment: breast, ovarian, and pancreatic, version 2.2021, NCCN clinical practice guidelines in oncology. J Natl Compr Cancer Netw. 2021;19:77‐102. 10.6004/jnccn.2021.0001 33406487

[23] Narod SA , Metcalfe K , Lynch HT , et al. Should all BRCA1 mutation carriers with stage I breast cancer receive chemotherapy? Breast Cancer Res Treat. 2013;138:273‐279. 23381743 10.1007/s10549-013-2429-x

